# Hyperhomocysteinemia results from and promotes hepatocellular carcinoma via CYP450 metabolism by CYP2J2 DNA methylation

**DOI:** 10.18632/oncotarget.14165

**Published:** 2016-12-24

**Authors:** Donghong Zhang, Jinli Lou, Xu Zhang, Lin Zhang, Fei Wang, Danfei Xu, Na Niu, Yidong Wang, Yue Wu, Wei Cui

**Affiliations:** ^1^ Department of Clinical Laboratory, Cancer Hospital, Chinese Academy of Medical Sciences, Beijing, 100021, China; ^2^ Department of Clinical Laboratory, Peking Union Medical College Hospital and Peking Union Medical College, Beijing, 100730, China; ^3^ Department of Genetics, Albert Einstein College of Medicine, Bronx, NY 10461, USA; ^4^ Department of Clinical Laboratory, Beijing You An Hospital, Capital Medical University, Beijing, 100069, China; ^5^ Department of Physiology and Pathophysiology, Tianjin Medical University, Tianjin, 300070, China; ^6^ Department of Pathology, The University of Texas MD Anderson Cancer Center, Houston, TX, 77030, USA

**Keywords:** hepatocellular carcinoma, homocysteine, CYP450, CYP2J2, DNA methylation

## Abstract

Hyperhomocysteinemia (HHcy) can result from liver disease or dysfunction and further alters intracellular lipid metabolism. Cytochrome P450 (CYP) arachidonic acid epoxygenases are expressed in human cancers and promote cancer metastasis. This study explored the interaction of Hcy and CYP450 metabolism in hepatocellular carcinoma (HCC). The levels of 4-epoxyeicosatrienoic acid (EET) isomers and their generative enzyme CYP2J2 level as well as intracellular Hcy level were higher in 42 cases of HCC than in paired non-tumor tissue. Elevated Hcy-decreased DNA methylation on SP1/AP1 binding motifs and enhancement on the CYP2J2 promoter via ERK1/2 signaling was essential for CYP2J2 upregulation and EET metabolism. Increased Hcy level enhanced the neoplastic cellular phenotype, which was reversed by CYP2J2 knockdown *in vitro*. Furthermore, tumor growth and size as well as patterns of CYP2J2 expression and DNA demethylation were increased with HHcy in mice induced orthotopically by 2% (wt/wt) L-methionine with or without folate deficiency. Moreover, the effect was attenuated by shRNA knockdown of CYP2J2. Thus, HHcy results from but can also promote hepatocarcingenesis via CYP450-EET metabolism by crosstalk of DNA demethylation of CYP2J2 and ERK1/2 signaling.

## INTRODUCTION

Hepatocellular carcinoma (HCC) is the fifth most frequently diagnosed cancer worldwide in men and the seventh in women and the second most frequent cause of cancer deaths; about half of these cases and deaths occur in China [1]. China ranked 12^th^ in 1990 of life lost due to HCC among all diseases and 6th in 2010 [2]. The emergence of metabolomics focusing on the concentrations and fluxes of low-molecular-weight metabolites (<~1000 m/z) in biofluids provides a powerful approach to identify molecule biomarkers and understand the mechanism of occurrence and progression of cancers, including HCC [3].

Homocysteine (Hcy) is a nonprotein, sulfur-containing amino acid that can be remethylated to methionine or catabolized, mainly in the liver. Increasing evidence suggests that rapidly proliferating tumor cells show elevated level of circulating Hcy [4], including in HCC [5]. In HCC, the Hcy level gradually increases from healthy tissue to liver cirrhosis and carcinoma tissue [6]. This finding might be related to depleted folate and inactivated methionine synthase in cells as well as mutation of methylenetetrahydrofolate reductase, which would induce lack of conversion of Hcy to methionine and its accumulation [5, 7]. The elevated Hcy level could specifically convert S-adenosyl homocysteine (SAH), a competitive inhibitor of most methyltransferases, further inducing DNA hypomethylation [8], which represents an epigenetic mechanism in carcinogenesis. Previous studies indicated that Hcy could promote the expression of cyclin A [9], platelet-derived growth factors [10], fibroblast growth factor 2 [11] and P66shc [12] via DNA hypomethylation in vascular endothelial cells [13].

Elevated Hcy level may result from but also explain HCC. Hyperhomocysteinemia (HHcy) alters intracellular lipid metabolism [13]. Lipid autacoids such as prostaglandins, leukotrienes, epoxyeicosatrienoic acids (EETs) and hydroxyeicosatetraenoic acids produced from arachidonic acid (AA) by cyclooxygenase (COX), lipoxygenase (LOX) and cytochrome P450 (CYP) pathways are abundant in the phospholipids of cellular membranes. COX and LOX but not CYP pathways have been fully characterized in carcinogenesis [14–16]. Liver has 4 regioisomeric EETs (5,6-, 8,9-, 11,12-, and 14,15-EET) that are mainly released by CYP2C8, -2C9 and -2J2 from AA; they are then further metabolized by soluble epoxide hydrolase (sEH) to dihydroxyeicosatrienoic acids (DHETs), which are considered less active than EETs. The Wang laboratory [15, 17] performed the initial pioneering studies demonstrating that CYP epoxygenases can promote cancer growth and metastasis, which was further confirmed by others [18, 19]. We previously demonstrated that DNA methylation of the sEH promoter inhibited its expression by an SP1-dependent mechanism in HepG2 cells [20].

Despite many studies characterizing the pro-angiogenic and anti-inflammatory role of the COX, LOX and CYP450 pathways in cancers, the metabolic analysis of AA metabolites and regulation of CYP450 enzymes in HCC remain poorly studied. Here, we collected 42 samples of HCC tissue and paired adjacent noncancerous tissue and explored the characteristics of AA metabolism by liquid chromatography-mass spectrometry (LC-MS/MS). Specifically, we found *in vitro* and *in vivo* that increased Hcy level could promote CYP2J2 expression and EET metabolism via DNA demethylation and the mitogen-activated protein kinase (MAPK) pathway deletion to further increase the risk of HCC.

## RESULTS

### Elevated intracellular Hcy level is associated with CYP2J2 upregulation and EET metabolism in HCC

**Figure 1:**
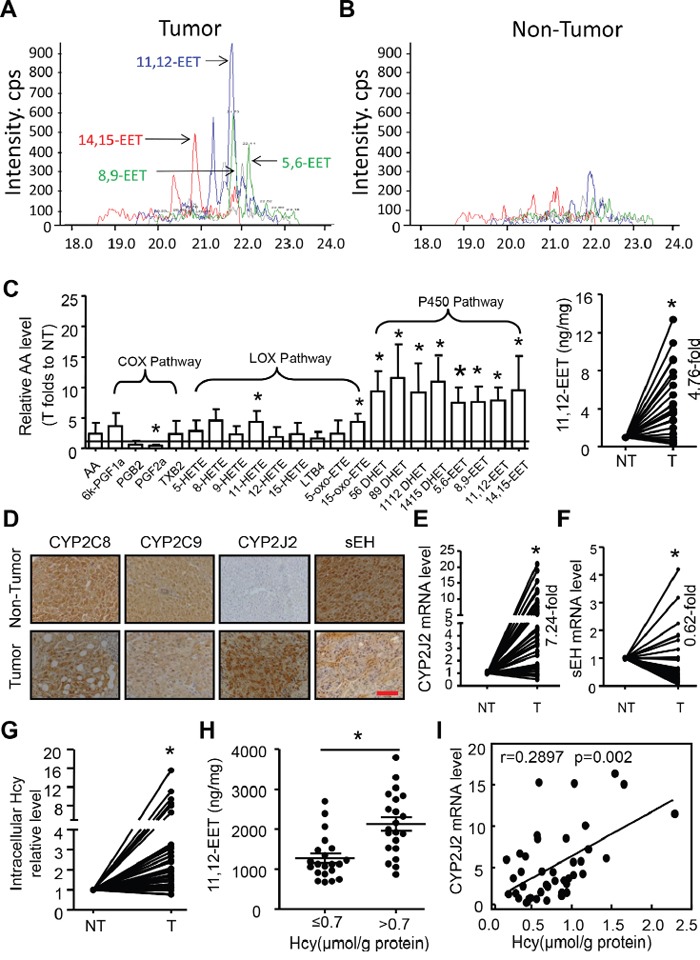
Elevated CYP2J2 expression and epoxyeicosatrienoic acid (EET) metabolism associated with homocysteine (Hcy) level in hepatocellular carcinoma (HCC) **A, B**. Representative chromatograms of 4 regioisomeric epoxyeicosatrienoic acids (EETs) in hepatocellular carcinoma (HCC) tumor and adjacent non-tumor tissue by LC/MS/MS assay. **C**. Left panel: Relative fold changes of 22 defined AA metabolites in 8 HCC tumor (T) and adjacent non-tumor (NT) tissue samples by LC/MS/MS assay. Right panel: Relative fold change of 11,12-EET levels in 42 pairs of HCC tumor (T) and adjacent non-tumor (NT) tissue by ELISA. **D**. Representative immunohistochemical staining of CYP2C8, CYP2C9, CYP2J2 and sEH in cross-sections of tumor and non-tumor tissue. Scale bars, 50 μm. **E, F**. qRT-PCR analysis of the relative mRNA expression of CYP2J2 and sEH in 42 cases of HCC tumor (T) and adjacent non-tumor (NT) tissue. β-actin expression was a normalization control. mRNA level of CYP2J2 and sEH in NT tissue was set to 1 for each paired HCC tumor tissue. **G**. The intercellular levels of Hcy in HCC tumor (T) and non-tumor (NT) tissue. **H**. 11,12-EET level in intracellular Hcy in HCC tissue. **I**. Correlation of Hcy concentration and CYP2J2 mRNA expression in HCC tissue. Data are mean±SD. *P<0.05 vs. NT.

To specifically analyse the quantity of AA metabolites in HCC, we screened AA and metabolites in 8 HCC tumor and adjacent non-tumor tissue samples by LC-MS/MS. Only 22 of 80 putative abundant AA metabolites were detectable in HCC and were further analysed (Figure 1A-1C). AA was the most abundant in tumor tissues, about 721.04±358.32 ng/mg, followed by P450_19-HETE and LOX_15-HETE. Importantly, the abundance of metabolites in the CYP450 pathway but not COX or LOX pathways was higher in tumor than adjacent non-tumor tissue: 5,6-EET, 8,9-EET, 11,12-EET, 14,15-EET and their corresponding DHETs. Of note, 11,12-EET level showed a 4.7-fold increase in 42 cases of HCC tissues compared with paired adjacent non-tumor tissue samples.

This evidence prompted us to examine the expression of enzymes of EET synthesis and metabolites. CYP2C8 and CYP2C9 showed high expression (>70%) but no difference between HCC tumor and non-tumor tissue. The mRNA and protein levels of CYP2J2 were higher in tumor than non-tumor tissue, but sEH levels were less (Figure 1D-1F, Supplementary Table 1), which might promote the synthesis and prevent metabolization of EETs. CYP2J2 protein levels were positively associated and sEH protein levels negatively associated with poorly differentiated tumor; as well, tumor sizes were greater and levels of alpha-fetoprotein were higher with high than low CYP2J2 level (Supplementary Table 2).

We previously performed a meta-analysis and found HCC risk associated with high serum Hcy level but low folate level [6]. Herein, we further found the intracellular Hcy level was higher in tumor than non-tumor tissue, but folate level was less (Figure 1G, Supplementary Figure 1A). Importantly, high intracellular Hcy level was frequently found associated with high levels of 11,12-EET (Figure 1H) and CYP2J2 (Supplementary Table 2). High Hcy level was moderately correlated with CYP2J2 mRNA level in HCC tissue (r=0.2897, Figure 1I) but negatively with FA level (r = −0.336; Supplementary Figure 1B). Therefore, elevated Hcy level in HCC tissue might participate in CYP2J2 expression and EET metabolism and increase the risk of HCC.

**Figure 2:**
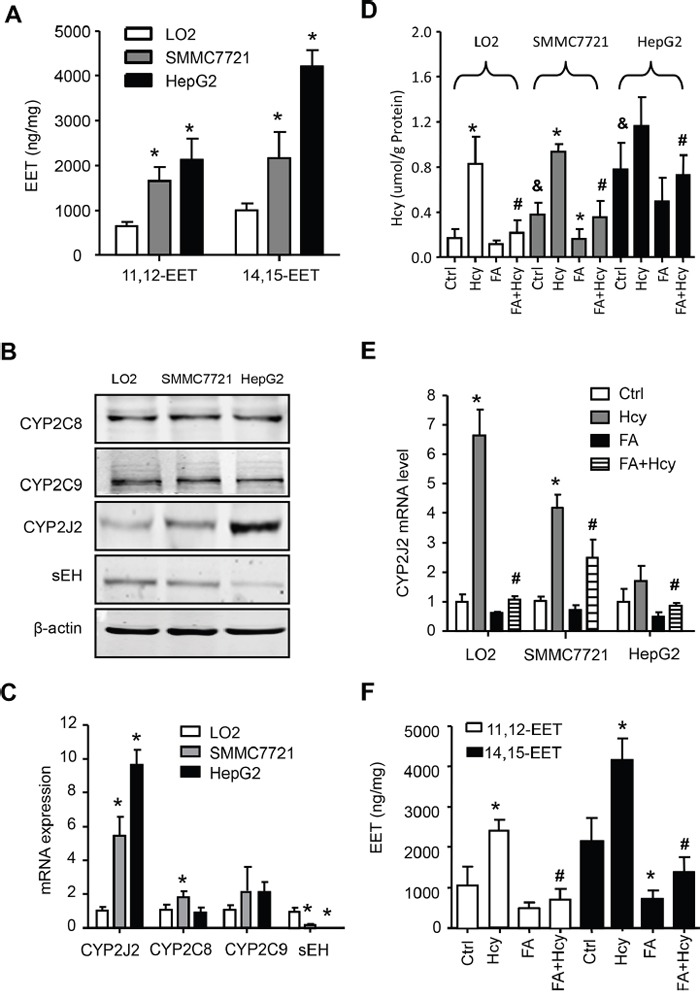
Hcy promoted EET secretion and CYP2J2 upregulation in HCC cells Intracellular levels of 11,12- and 14,15-EET **A**. and protein **B**. and mRNA **C**. levels of CYP2C8, CYP2C9, CYP2J2 and sEH by ELISA, western blot assay and quantitative RT-PCR, respectively, in LO2, SMMC7721 and HepG2 cells. Intercellular level of Hcy and folic acid (FA) **D**. and CYP2J2 mRNA **E**. in the above 3 cell lines, as well as 11,12-EET and 14,15-EET levels **F**. in SMMC7721 cells with Hcy and FA alone or combined. β-actin was an internal control. *P<0.05 vs. LO2 cells (A-C), * or ^#^P<0.05 vs. corresponding control or Hcy treatment; ^$^ P<0.05 vs. LO2 controls (D-F).

Clinical association does not prove causality. Here, we examined the EETs and their synthesis and metabolized enzyme levels in human liver cells, namely LO2 (normal liver), SMMC7721 (high differentiation of HCC) and HepG2 (low differentiation of HCC) cell lines. Level of 11,12-EET and 14,15-EET as well as CYP2J2 protein and mRNA levels were gradually increased, but sEH level was decreased in LO2, SMMC7721 to HepG2 cells (Figure 2A-2C). In addition, Hcy level was increased in LO2, SMMC7721 and HepG2 cells and was sensitive to treatment with Hcy and FA, especially in LO2 and SMMC7721 cells (Figure 2D). Next, we detected the effect of Hcy on EET metabolism: Hcy could increase the levels of CYP2J2 protein (Supplementary Figure 2A) and mRNA, and the level of 11,12-EET and 14,15-EET, which was reversed in part by pre-treatment with folic acid (Figure 2E-2F).

### Hcy-induced DNA demethylation facilitated CYP2J2 upregulation in HCC

**Figure 3:**
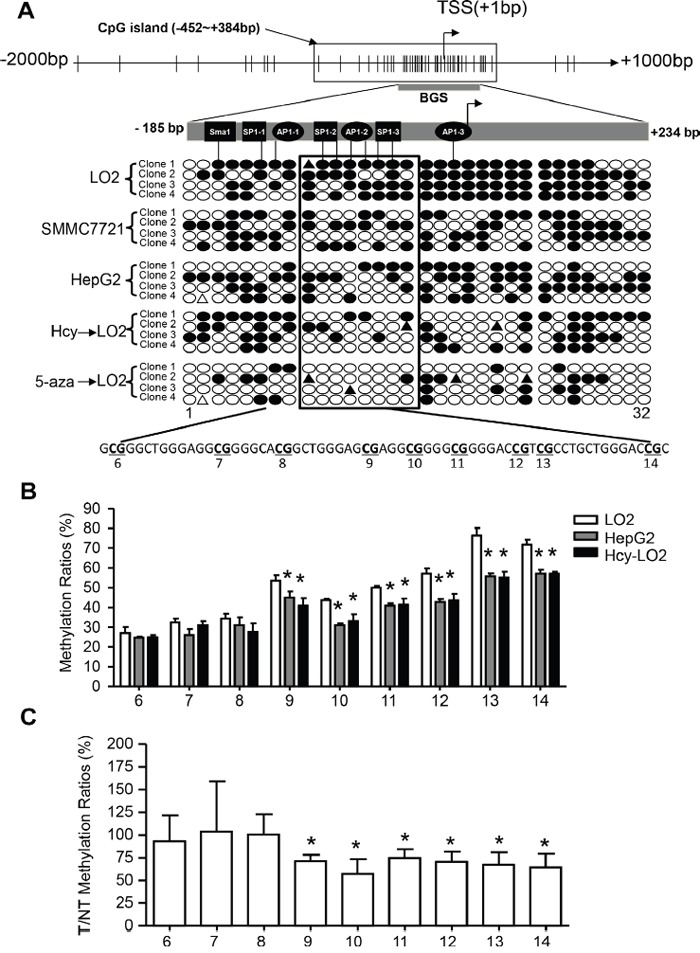
Hcy facilitated CYP2J2 upregulation in HCC cells by DNA demethylation **A**. Structure of CpG sites (short vertical lines), CpG island (box), bisulfite sequencing region (dark bar) and transcription start sites (TSS) on the CYP2J2 promoter. Bisulfate genomic sequencing (BGS) analysis of CYP2J2 in SMMC7721, HepG2 and LO2 cells treated with or without 50 μM Hcy and 4 μM 5-aza for 72 hours. Each row indicates a clone from BGS to obtain a representative sampling of 32 CpG methylation patterns in the core promoter. Each circle corresponds to a single CpG site. Methylated sites are shown as filled circles, unmethylated sites as empty circles, and deletion or mutation sites as filled triangles. **B, C**. Bisulfite pyrosequencing analysis comparing the mean methylation for each CpG site (6th-14th) on the CYP2J2 promoter in HepG2 and LO2 cells treated with or without Hcy and in 42 HCC tumor (T) and adjacent non-tumor (NT) tissue samples. Data are mean±SD. *P<0.05 vs. LO2 cells (B) or 100% (C).

The specific conversion of elevated Hcy level to SAH further inducing DNA hypomethylation represents a major mechanism [8]. Indeed, intercellular Hcy level was inversely correlated with 5-methylated cytosine level in HCC tissue (r = -0.346; Supplementary Figure 2B). We also found that Hcy treatment could mimic 5-aza, a DNA methyltransferase inhibitor, thereby promoting global DNA demethylation and upregulating CYP2J2 (Supplementary Figure 2A, 2C). To explore whether Hcy-induced epigenetic alteration activates CYP2J2 transcription, we used bioinformatics (http://www.urogene.org/cgi-bin/methprimer/methprimer.cgi) and analysed the sequence characteristics of the 5’-flanking region (-2000-+1000 bp) of the CYP2J2 gene (Figure 3A). We identified abundant CG dinucleotides and one CpG island spanning −452 to +384 bp. Furthermore, bisulfite sequencing assay revealed that DNA methylation of the CYP2J2 core promoter (−185 to +234 bp) changed from hypermethylated in LO2 cells (80.0%) to hypomethylated in SMMC7721 cells (51.6%) and HepG2 cells (46.9%). Treatment with Hcy or 5-aza in LO2 cells reduced DNA methylation to 39.0% and 17.2%. Specifically, the SP1-2, AP1-2 and SP1-3 binding sites on the bisulfite sequencing region (10-15^th^ CpG sites) were largely switched from methylated to unmethylated.

In addition, we performed bisulfite pyrosequencing to fully examine the DNA methylation status of the individual 6^th^ to 14^th^ CpG sites on the CYP2J2 promoter. Methylation ratios were decreased in the 9^th^ to 14^th^ CpG sites in HepG2 cells and Hcy-treated LO2 cells as compared with LO2 cells alone (Figure 3B). Similar decreased DNA methylation in this region was observed in the 42 HCC tissue samples versus adjacent non-tumor tissue (Figure 3C).

### Cooperation of SP1/AP1 binding sites was essential for Hcy-induced CYP2J2 promoter activity

**Figure 4:**
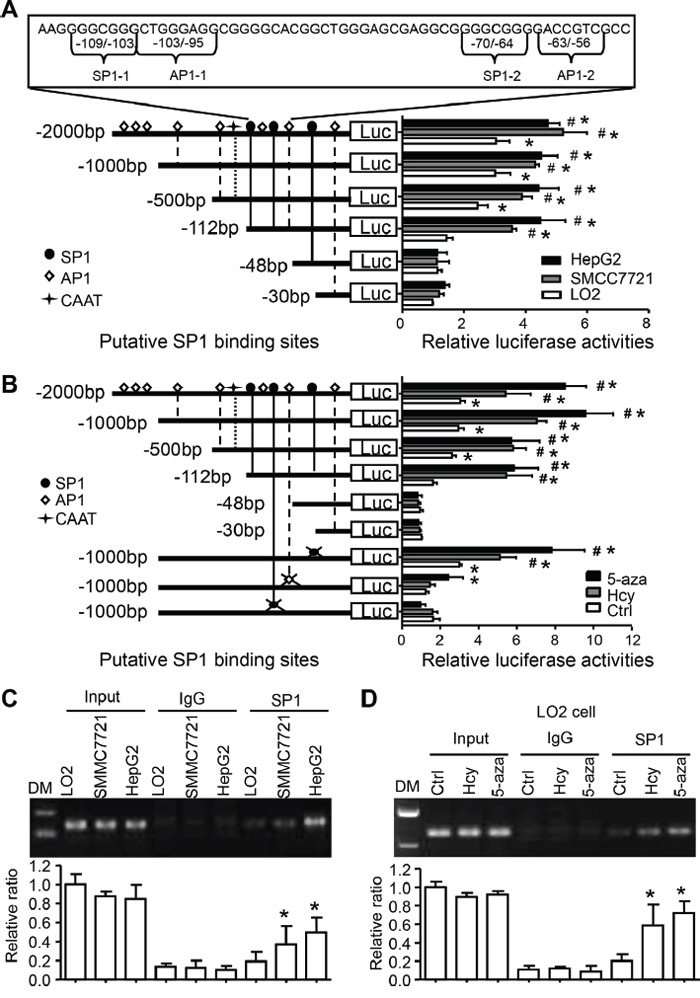
Hcy-reduced DNA methylation promotes SP1 binding and transcriptional activation of CYP2J2 **A**. The SP1 and AP1 binding sites are underlined in the human CYP2J2 sequence. Serial 5’-deletion constructs of CYP2J2 promoter were co-transfected with CMV–β-gal into LO2, SMMC7721 and HepG2 cells for luciferase induction assay. *P<0.05 vs CYP2J2 -48/+100 bp-Luciferase reporter vector (Luc) activity in LO2 cells with PBS treatment; ^#^P<0.05 vs luciferase activity of each construct in LO2 cells. **B**. AP1 or SP1 point-mutated constructs were generated on the basis of CYP2J2 -1000/+100 bp-Luc plasmids. Serial 5’-deletion constructs and mutated constructs were transiently co-transfected with CMV–β-gal (a transfection control) into LO2 cells and treated with Hcy or 5-aza for luciferase induction assay (relative to transfection with CYP2J2+30/−100 bp-Luc). *P<0.05 vs. CYP2J2 -48/+100 bp-Luc activity; ^#^P<0.05 vs. Ctrl at each constructs. **C**. ChIP assay with anti-SP1 antibody for immunoprecipitation in LO2 (treated with Hcy or 5-aza, **D.)** SMMC7721 and HepG2 cells; normal rabbit IgG was a control. Semi-quantitative PCR with CYP2J2 promoter-specific primers to detect the binding of SP1 to the CYP2J2 promoter region. Shows quantification of the binding ability. Data are mean±SD (n=3). *P<0.05 vs. LO2 cells (C) or non-tumor tissue (D).

Previous data showed that the c-Jun-responsive module in the 5’-flanking region of the human CYP2J2 gene was involved in its transactivation [21]. To further define the transcriptional activation of the effect of Hcy, serial deletions of the CYP2J2 promoter constructed in pGL3-basic plasmids for luciferase induction assays. The relative luciferase activity was strongly activated from -2000 to -112 bp of the CYP2J2 upstream sequence (containing SP1/AP1 binding sites) in SMMC7721 cells (3.6-fold) and HepG2 cells (4.5-fold) as compared with the -48/+100-bp construction (Figure 4A). However, this pattern was only slightly activated in LO2 cells. The 3 cell types showed no response to deletion of -30 to -48 bp of the CYP2J2 upstream sequence. In addition, 5-aza or Hcy treatment could augment CYP2J2 transcription activity by 5- to 10-fold in the region containing SP1-2/SP1-2 binding sites in LO2 cells (Figure 4B). This effect was not found on mutation of the SP1-2 and/or SP1-2 binding sites in SMMC7721 or HepG2 cells (data not shown). Indeed, the mutation of the AP1-2/SP1-2 binding sites decreased the promoter activities at the basal level in response to 5-aza or Hcy, with no effect with mutation of the -41~-39–bp site (Figure 4B). Thus, the region between -112 and -48 bp (containing AP1-2/SP1-2 binding sites) seemed to be responsible for AP1/SP1-dependent transactivation with Hcy treatment or DNA methylation in HCC.

**Figure 5:**
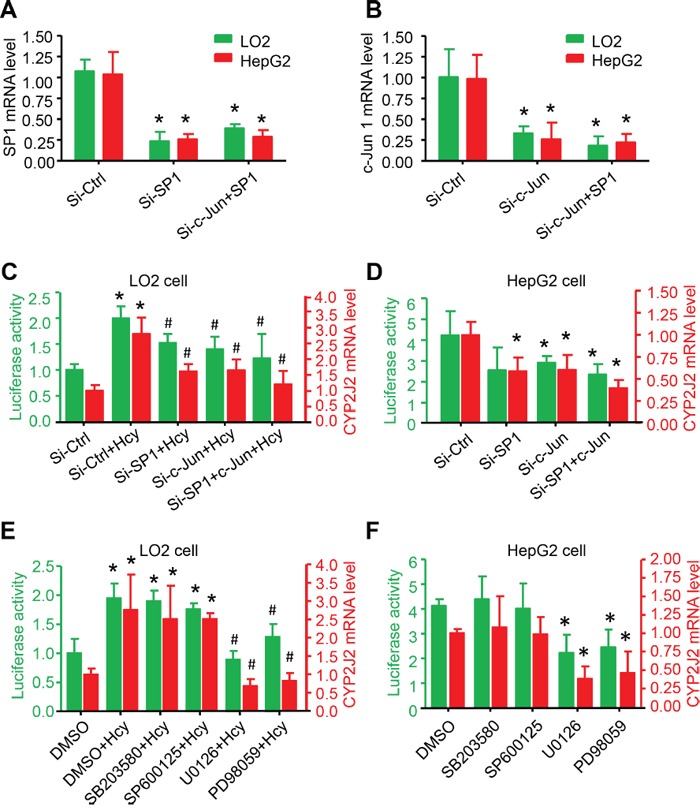
ERK1/2 signalling pathway participated in Hcy upregulating CYP2J2 promoter activity and expression qRT-PCR and relative luciferase activity assay of SP1 **A**., c-Jun **B**. and CYP2J2 mRNA expression and transcriptional activity (CYP2J2+1000/−100 bp-Luciferase reporter vector), respectively, with 40-nM siRNA targeting SP1 and c-Jun for 48 h: treatment with MAPK inhibitors (PD98059, U0126, SP600125 and SB203580) for 1 h with or without Hcy treatment in LO2 cells **C, E**. and HepG2 **D, F**. cells. β-actin was an internal control for qRT-PCR assay. Data are mean±SD (n=3). *P<0.05 vs. Ctrl; ^#^P<0.05 vs. Hcy.

We further performed ChIP assays to test whether SP1 increases its binding to the CYP2J2 promoter in response to Hcy-induced DNA demethylation. The direct binding of SP1 to the CYP2J2 proximal promoter region (−161 to −22 bp) was strongly enhanced in SMMC7721 cells (2.20±0.44-fold, p=0.045) and HepG2 cells (2.55±0.13-fold, p=0.002) and on treatment with Hcy (3.72±1.43-fold, p=0.031) or 5-aza (4.11±1.13-fold, p=0.012) in LO2 cells (Figure 4C-4D).

c-Jun, one of the two heterodimers of AP1 bound to the -105/−88 region, has a major role in CYP2J2 regulation [21]. To further investigate the cooperation of SP1 and c-Jun in CYP2J2 regulation, we knocked down SP1 or c-Jun in LO2 cells by 8-h siRNA transfection (Figure 5A-5B). The Hcy-induced CYP2J2 promoter activity and mRNA expression were significantly reduced with knockdown of SP1 and c-Jun alone or together in LO2 cells (Figure 5C). In addition, deletion of SP1 and c-Jun alone or together could directly decrease the basal level of CYP2J2 promoter activity and mRNA expression in HepG2 cells (Figure 5D).

### ERK1/2 signalling pathway promoted CYP2J2 promoter activity and expression

The phosphorylation of the mitogen-activated protein kinase (MAPK) superfamily increases rapidly after Hcy stimulation and is further involved in gene expression and cellular functions in endothelial cells [22]. To explore which MAPK leads to CYP2J2 transcription via Hcy in HCC, we incubated LO2 cells with 20 μM of specific inhibitors for ERK1/2 (PD98059 and U0126), JNK (SP600125), or p38 (SB203580) for 1 h before stimulation with Hcy. Hcy-induced CYP2J2 promoter activity and mRNA expression were significantly inhibited by U0126 or PD98059 but not SB203580 or SP600125 in LO2 cells (Figure 5E). U0126 or PD98059 significantly and directly inhibited CYP2J2 promoter activity and mRNA expression in HepG2 cells (Figure 5F). Thus, transactivation of the CYP2J2 promoter requires phosphorylation of ERK1/2 but not p38 MAPK and JNK.

### CYP2J2 knockdown inhibited oncogenic activity of Hcy *in vitro*

**Figure 6:**
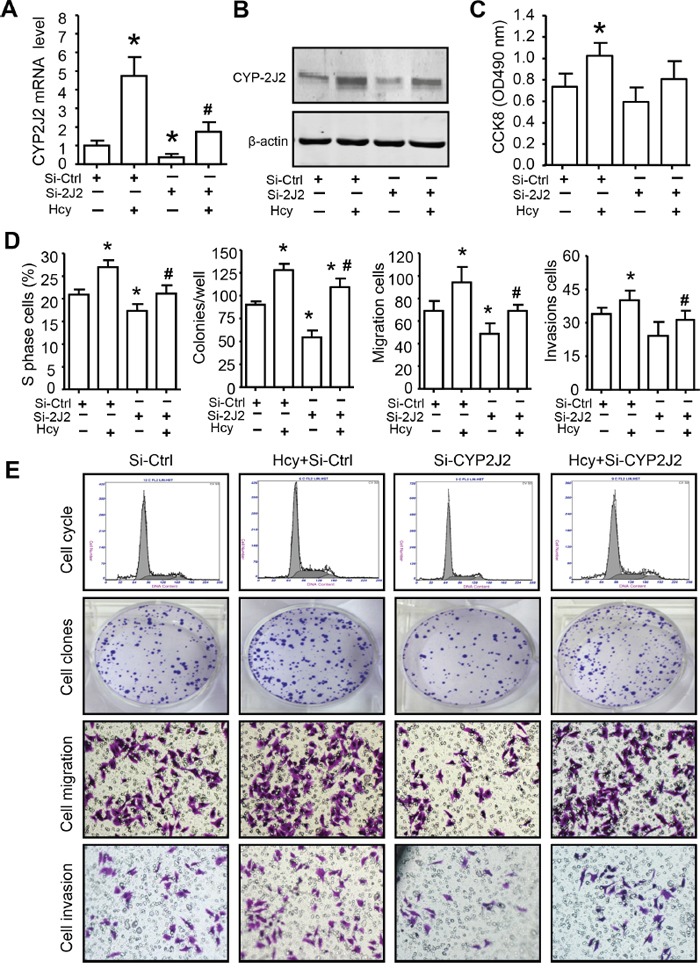
CYP2J2 involved in Hcy promotes aggressive human HCC *in vitro* qRT-PCR **A**. and western blot **B**. assay of the mRNA and protein expression, respectively, of CYP2J2 in SMMC7721 cells with siRNA knockdown of CYP2J2 (Si-2J2) or control (Si-Ctrl) with or without Hcy treatment. **C-E**. The quantify of cell proliferation, clonogenicity, migration and invasion of SMMC7721 cells were measured by CCK-8 assay, flow cytometry, colony formation, tranwell assay, respectively. Data are mean±SD (n=3). *P<0.05 vs. Si-Ctrl; ^#^P<0.05 vs. Hcy.

CYP2J2 and EETs have been implicated in promoting the neoplastic cellular phenotype including cell proliferation and migration [17, 18, 23]. Hcy increased CYP2J2 expression in SMMC7721 cells, which was significantly inhibited by pre-transfection with an siRNA target for CYP2J2 for 8 h (Figure 6A-6B). MTT assay, flow cytometry, colony formation and tranwell assay revealed that Hcy treatment promoted SMMC7721 oncogenic activity, including cell viability, cell cycle, clonogenicity, cell migration and invasion (Figure 6C-6E). CYP2J2 knockdown could significantly reverse the effect of Hcy on cell proliferation (66.82±10.14% for MTT, 78.48±6.67% for clonogenicity and 85.35±7.45% for cell cycle), migration (81.20±10.69%) and invasion (73.13±5.81%).

### Hcy-mediated delivery of CYP2J2 promoted tumorigenesis *in vivo*

**Figure 7:**
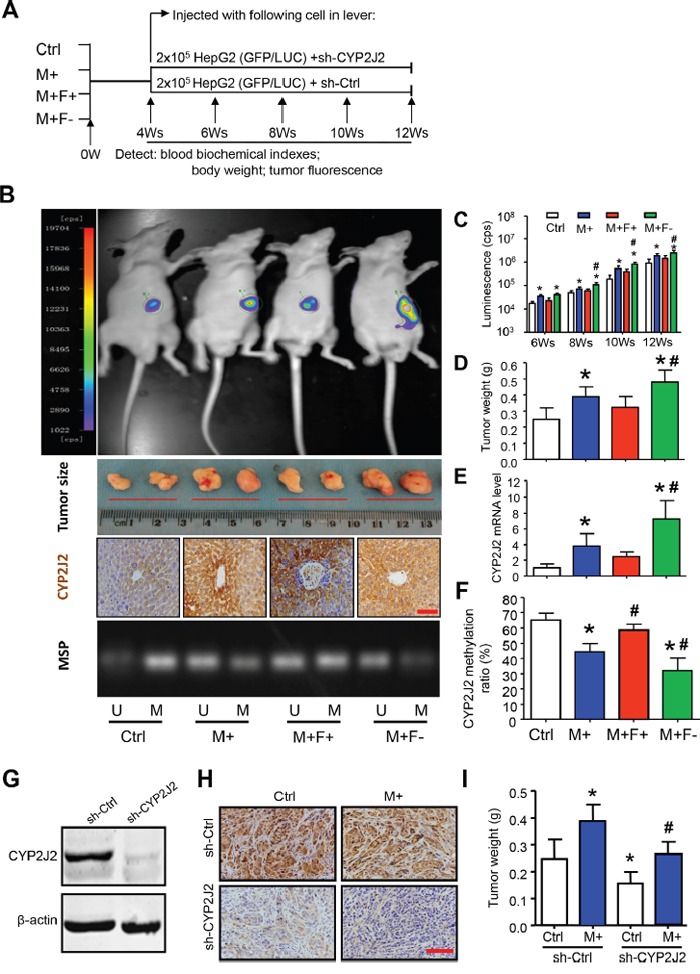
Hcy promotes hepatocellular tumorigenesis by CYP2J2 *in vivo* **A**. Targeting strategy and timeline of Hcy delivery in an orthotopic mouse model of HCC. **B**. Representative images of luciferase activity by the IVIS imaging system, tumor formation, and immunohistochemical staining for CYP2J2 at 8 weeks after luciferase-tagged HepG2 cells were injected into the left liver lobe of nude mice with different diets. **C**. Luminescence (cps) at 8, 10, 12, and 14 weeks. **D-F**. tumor weight (g) and CYP2J2 mRNA and protein expression. Methylation-specific PCR (MSP) analysis and ratio of DNA methylation to total methylation and unmethylation in liver of nude mice with different diets for 8 weeks. M: methylated, U: unmethylated. **G**. Western blot analysis of CYP2J2 protein expression with stable transfection with short hairpin CYP2J2 (sh-CYP2J2) and sh-Ctrl in HepG2 cells. Representative images of immunohistochemical staining (400x) of CYP2J2 **H**. and tumor weight **I**. at 8 weeks after HepG2 cells were transfected with sh-CYP2J2 and sh-Ctrl and injected into the left liver lobe of nude mice with Ctrl and M+ diet. Data are mean ± SD (n=8). *P<0.05 vs. Ctrl; ^#^P<0.05 vs. M+. Scale bars, 50 μm.

Finally, we aimed to investigate whether Hcy-mediated CYP2J2 might drive tumorigenesis *in vivo* by using a bioluminescence imaging system for sensitive detection of tumor growth in a mouse model of orthotopically induced HCC (Figure 7A). As compared with the control diet, the AIN-93G diet supplemented with methionine (M+) significantly increased serum levels of Hcy, total bile acids, total cholesterol, low-density lipoprotein and C-reactive protein (CRP); as compared with the M+ diets, folate deficiency (M+F- diet) or enrichment (M+F+ diet) significantly increased or decreased serum Hcy and CRP levels, respectively (Supplementary Table 3). Bioluminescence assay demonstrated a significant increase in tumor growth rate and size with the M+ diet as compared with the control diet (Figure 7B, 7C). These observations were further confirmed by measuring tumor weight (Figure 7D). Furthermore, immunostaining, qRT-PCR and MSP assay revealed the increased pattern of CYP2J2 protein and mRNA level and DNA demethylation ratios (focus on SP1-2/AP1-2 motif), respectively, in livers of mice with the M+ diet (Figure 7B, 7E, 7F). Tumors with the M+ diet were poorly differentiated HCCs, often with more than a two-cell–thick trabecular or pseudoglandular pattern, occasionally with liver metastasis and necrosis (Supplementary Figure 3). Furthermore, CYP2J2 expression was lower and tumors were smaller in mice with than without CYP2J2 knockdown in control and M+ diet groups, respectively (Figure 7G-7I).

## DISCUSSION

HCC is accompanied by metabolic dysfunction in the liver that reflects changes in gene expression, protein secretion, and small metabolite concentrations [3, 24]. Our study linked two main AA and methionine metabolites by an epigenetic mechanism. We first used tissue metabolomics to examine the changes in abundance of 80 AA metabolites and found increasing levels of 4 isomers of EETs and their corresponding diols but not metabolites in the COX or LOX pathway in HCC tissue as compared with adjacent non-tumor tissue. The elevated intracellular Hcy-Induced cooperation of DNA demethylation and ERK1/2 signalling pathway on SP1/AP1 binding sites were essential for CYP2J2 upregulation, which contributed to EET metabolism and increased the risk of HCC. As well, Hcy-mediated delivery of CYP2J2 promoted tumorigenesis *in vivo* and *in vitro*. Our study highlights the interaction of the physiological process of Hcy and AA metabolism at the molecular level in HCC progression. Such an understanding could facilitate new therapeutic approaches to Hcy caused by HCC and metabolic dysregulation.

The causes of HCC are heterogeneous, with metabolic, genetic and epigenetic alterations associated with tumor progression. Uniquely, the methionine synthesis pathway is central for intersecting the above 3 mechanisms in the liver [25]. Recent studies indicated that the development of cirrhosis and HCC involves folate deficiency, mutation of methylenetetrahydrofolate reductase and loss of expression of key enzymes involved in the hepatic one-carbon cycle [26–28], for combined elevated level of Hcy and S-adenosyl homocysteine (SAH) in cells and circulation. However, Hcy and SAH are pathologic consequences and also a major cause of tumor progression [29], chronic renal failure [30], Alzheimer disease [31] and even vascular disease [32]. Our study with cell culture and mouse models confirmed that HHcy level could promote the oncogenic activity of HCC, such as cell proliferation, colony formation, migration and invasion.

Tissue metabolomic approaches are being widely used for the discovery of new biomarkers or therapeutic targets for clinical applications and investigation of the carcinogenesis mechanism, including in HCC [24]. Here, we screened and validated the increased levels of 4 regioisomeric EETs and their corresponding DHETs but not metabolites in the COX and LOX pathway in HCC tumor tissue. Transcriptional upregulation of CYP2J2 and downregulation of sEH in HCC might explain the EET secretion and accumulation, related to the differentiation of tumor, tumor size and increased level of alpha-fetoprotein as well as intracellular Hcy level. Increasing evidence indicates that CYP450 epoxygenases and their key enzymes are involved in cancer progression [18]. High levels of EETs have been detected in urine, blood and tissue from some cancer patients [17, 22, 33]. As well, both CYP2J2 overexpression and EETs treatment in cancer cells could stimulate cell proliferation, survival, migration, and invasion [17, 33, 34]. Consistent with other studies, our clinical data indicated that elevated Hcy level in HCC might participate in CYP2J2 expression and EET metabolization and increase the risk of HCC.

We previously demonstrated that SP1 was involved in the decreased transcription of sEH as a result of DNA methylation in HepG2 cells, which might contribute to epigenetic mechanism-induced carcinogenesis in hepatocytes [20]. Here, reduced sEH level might explain the decreased enzymatic hydroxylation of EETs to their corresponding DHETs. Similar to the sEH gene promoter, a typical CpG island region in the CYP2J2 promoter suggested that DNA methylation may also be involved in transcriptional regulation. The difference is demethylated CYP2J2 but methylated sEH, related to their reverse expression patterns in HCC. Indeed, the CYP2J2 promoter changed from methylated to unmethylated with Hcy treatment, which was reversed by folic-acid pre-treatment in normal liver cells or high-differentiated but not low-differentiated HCC cells. A similar pattern was seen in noncancerous and HCC tissue. The c-Jun and AP1 conjunction-responsive module between -112 and -50 bp in the CYP2J2 proximal promoter has a major role in transactivational CYP2J2 expression [35]. The change in methylation might result from Hcy-induced CYP2J2 promoter demethylation.

Hcy could stimulate the activation of mesangial-cell signal transduction of MAPKs, including ERK1/2, JNK, and P38, known as the acute response to hormones [35]. SP1 and AP1 are often the final targets of signal-transducing kinase cascades via their activation and bind to their respective target promoters and trigger expression of the corresponding genes [36]. In ECs, Hcy-induced JNK phosphorylation and SP1 and AP1 activation is involved in stromal cell-derived factor 1 (SDF-1) expression [37]. We found that Hcy-induced ERK1/2 but not JNK and P38 signalling cooperated with SP1/AP1 activity and was involved in Hcy-induced DNA demethylation on the CYP2J2 promoter in hepatocellular carcinogenesis, which could explain the increased level of EETs.

The efficacy of the Hcy-promoted hepatocellular carcinogenesis by CYP2J2 DNA demethylation was demonstrated *in vivo*. We first established a mild and moderate HHcy mouse model for 4 weeks and injected a low number of HepG2 cells to establish chronic HCC in mice for another 8 weeks. Tumor weight was positively associated with serum Hcy level. In contrast, this effect could be rescued with CYP2J2 gene in injected HepG2 cells. Together, these data are consistent with our *in vitro* findings and suggest that inhibiting CYP2J2 expression may protect against Hcy-promoted tumorigenesis and injured liver.

**Figure 8:**
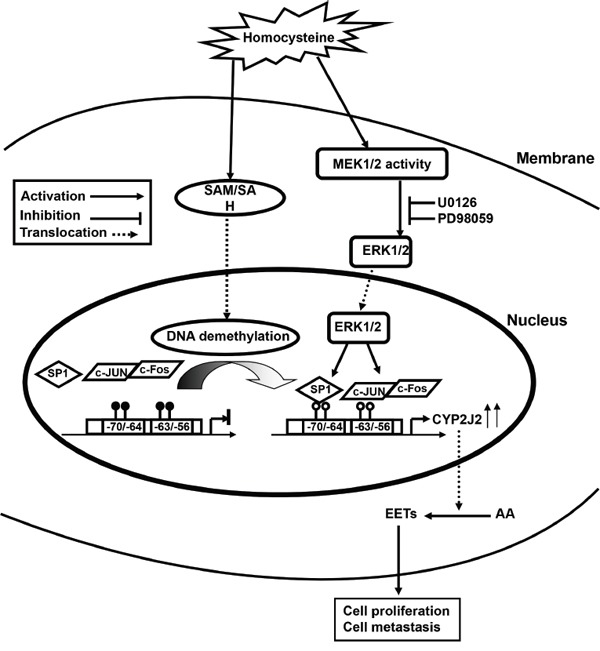
Possible mechanism of Hcy-facilitated hepatocellular carcinogenesis by CYP2J2 transcriptional regulation Elevated Hcy level induces the accumulation of S-adenosyl homocysteine (SAH), which demethylates SP1 and AP1 (c-Jun/c-Fos) binding motifs on the CYP2J2 promoter via the ERK1/2 pathway. Combined effects increase CYP2J2 transcriptional activation and endogenous EET synthesis and promote primary tumor growth and metastasis.

In summary, our study demonstrates that both Hcy and AA metabolism show dynamic change but also interact in HCC progression. Hcy stimulated the cooperation of SP1/AP1 activity by the ERK1/2 pathway, and DNA demethylation on the CYP2J2 promoter was involved in transcriptional upregulation of CYP2J2, which contributed to increased level of EETs in HCC (Figure 8). The methionine synthesis pathway could be improved by supplementation with folic acid to inhibit the metabolism of the CYP450 pathway by DNA methylation at the early stage of HCC. Our findings warrant a future larger cohort study to validate the present observations.

## MATERIALS AND METHODS

### Tumor specimens

42 cases of HCC clinical tumor tissues and their paired non-tumor tissues were collected from HCC patients undergoing hepatectomy in the Department of Surgery, You’an Hospital, University of Capital Medical College. All histology diagnoses for HCC were reviewed by two senior pathologists. Tissue samples were immediately snap-frozen in liquid nitrogen and stored at -80°C. The study protocol conformed to the ethical guidelines of the 1975 Declaration of Helsinki and approved by the ethics committee of University of Capital Medical College and Peking Union Medical College Hospital/Chinese Academy of Medical Sciences, and patients gave their informed consent to be in the study.

### Cell lines

Normal liver (LO2) and the HCC cell lines (SMMC7721, HepG-2 and HepG2-GFP) were obtained from China infrastructure of cell line resources (Beijing) and validated within 6 months by short tandem repeat (STR) analysis using the AmpFlSTR Identifiler kit (Applied Biosystems). The STR profiles matched with known ATCC fingerprints (ATCC.org). Cells were grown in Dulbecco's modified Eagle medium (DMEM, Gibco). All media was supplemented with 10% fetal bovine serum (Highclone) and 1% penicillin/streptomycin, and cell lines were maintained at 37°C in a humidified atmosphere under 20% O_2_ and 5% CO_2_.

### EET/DHET detection

Episomes of 11,12- and 14,15-EET in cells were determined by use of an ELISA kit (R&D, USA) [38]. Briefly, tissue protein was collected in triphenylphosphine, acidified and extracted with ethyl acetate, then organic phases were collected and evaporated. Sediment was dissolved in ethanol, and secretion of DHET was measured. The episomal DHET level was measured without hydrolysis of EET in the same sample and subtracted from total DHET level to obtain the EET level in the sample. All EET/DHET analyses were performed in biological triplicate for each sample.

### Quantitative reverse transcription PCR (qRT-PCR)

Total RNA was isolated from the indicated tissues or cell lines by use of Trizol (Invitrogen, CA, USA) per the manufacturer's protocol. cDNA was synthesized from 0.5 μg total RNA by using the SuperScript strand synthesis system (Invitrogen, CA, USA). qRT-PCR involved use of LightCycler480 (Roche, Switzerland). β-actin expression was a normalization control, and water was a negative control for mRNA quality and quantity. Relative changes in expression levels were calculated by the 2^-ΔΔ^CT method.

### Western blot analysis

Cell lysates were separated on 10% SDS-PAGE and transferred to nitrocellulose membranes, which were incubated with the primary antibodies: CYP2J2 (Abgent, AP7450c, 1:500), CYP2C8 (Proteintech, 16546-1-AP, 1:1000), CYP2C9 (Bioss, bs-2887R, 1:1000), sEH (sc-25797, 1:500) and β-actin (both Santa Cruz Biotechnology, sc-8432, 1:5000) overnight at 4°C, then with goat anti-mouse IgG antibody IRDye600CW (Li-Cor, Lincoln, NE, 1:2000) for 1 hour at room-temperature. Membrane bands were quantified by use of the Odyssey infrared imaging system (LI-COR). The relative expression was quantified and normalized to that of β-actin by use of ImageJ (US National Institutes of Health).

### Cell proliferation, migration and invasion assay

For cell proliferation assay, we used the colony-formation assay: cells were plated onto 6-well plates and cultured for 14 days. The number of colonies was counted after fixation. For flow cytometry, cells were harvested, fixed and re-suspended in phosphate buffered saline (PBS) containing propidium iodide (PI) and RNase A. Cell cycle profiles of 1× 10^4^ cells were analysed by use of a FACS Calibur flow cytometer (Becton Dickinson, Franklin Lakes, NJ). For 4,5-dimethyl-2-thiazolyl)-2,5-diphenyl-2-H-tetrazolium bromide (MTT) assay, the Cell Counting Kit-8 (CCK8, Sigma, 96992) was used: cells seeded on 96-well plates were stained for various times with 20 μl CCK8. Absorbance was measured at 450 nm wavelength. For cell migration and invasion assays, cells were seeded on the upper chamber of a Transwell or a Matrigel-coated Transwell chamber (BD Biosciences, NJ) in 24-well plates with 8.0-mm pores (Corning) in serum-free media. The lower chamber contained DMEM with 10% fetal bovine serum as a chemoattractant. After 24 h of incubation, the nonmigrated or noninvaded cells were gently removed from the upper chamber with use of a cotton swab. Cells were fixed and stained with Giemsa solution and counted in 5 randomly chosen visual fields.

### Immunohistochemistry

Immunohistochemistry was performed as described [39] with the following primary antibodies overnight at 4°C: CYP2J2, CYP2C8, CYP2C9 and sEH, which origin were as western blot assay, but with 1:200 dilution. PBS was used as a negative control. Slides were incubated with polyclonal peroxidase-anti-mouse/rabbit IgG antibody (PV9000, Zymed Laboratories) for 1 hour at room-temperature and counterstained lightly with hematoxylin. The immunostaining was scored separately by 2 independent gastrointestinal pathologists (N.N and J.L.L.), who were blinded to the histopathologic features and patient data of the samples; the scoring of positive immunoreactivity was as described previously [21, 40].

### Construction of reporter plasmids for human CYP2J2 promoter and luciferase activity assay

A human CYP2J2 3’-UTR (-2000/+100 bp) fragment was isolated from a human genomic library and cloned into the *XhoI* and *NotI* sites of the PGL-3-basic luciferase reporter vector. A series of CYP2J2-promoter deletion constructs was created and amplified by PCR. For mutations of the putative AP1/SP1–like elements, binding sites were generated from the CYP2J2-1000/+100-bp construct by using the TaKaRa MutanBEST Kit (TaKaRa Biotechnology, China). All constructs were confirmed by sequencing. Cells were co-transfected with the corresponding reporter plasmid and CMV–β-gal plasmid (to normalize for transfection efficiency) in each experiment according to the manufacturer's instructions. The dual-luciferase reporter assay (Promega, E2231) was performed after 24 h in accordance with the manufacturer's instructions.

### siRNA knockdown and transfection

The siRNA targets CYP2J2, c-Jun and SP1, short hairpin RNA (sh-RNA) target CYP2J2, and corresponding controls were designed and synthesized by GenePharma (Shanghai). ShRNA was cloned into the FseI site of scAAV.EF1α.eGFP to produce scAAV. According to the manufacturer's instructions for jetPEI (Polyplus, San Marcos, CA), siRNAs at 50 nM were transiently transfected into HepG2 or LO2 cells and harvested at the indicated times. The transfection efficiency of gene knockdown was confirmed by qRT-PCR or western blot assay.

### Chromatin immunoprecipitation (ChIP) assay

ChIP assay was performed as we previously described [10, 20, 41]. In brief, cells were fixed, sonicated, and immunoprecipitated (IP) with polyclonal antibodies for SP1 (Abcam, ab133596) or IgG (Santa Cruz Biotechnology, sc-2027) over night at 4°C. IP-complexed DNA was extracted and underwent PCR amplification for SP1 binding sites on the CYP2J2 promoter. The resulting DNA was resolved on 1.5% agarose gel and quantified by densitometry by use of ImageJ with normalization to the input control.

### Animal model

Male BALB/c-nu nude mice at 4 weeks of age were divided into 4 groups for treatment: control (n=16); standard AIN-93G diet, M+ (n=16), AIN-93G diet supplemented with (wt/wt) L-methionine (Sigma, USA); M+F- (n=8), M+ diet deficient in folate, vitamin B-6, and vitamin B-12; and M+F+ (n=8) diet supplemented with these B vitamins. These diets were as described [42] with modification. After 8 weeks of diet, 2×10^5^ sorted HepG2-GFP-luciferase–shCtrl cells were injected in the left liver lobe of the 4 groups (n=8, each). Another set of Ctrl and M+ groups (n=8, each) were injected in the left liver lobe with HepG2-GFP-luciferase–shCYP2J2 cells. We established CYP2J2 gene knockdown in HepG2 cells with GFP/LUC by transfection with sh-CYP2J2 or sh-Ctrl, and positive clones were selected with DMEM supplemented with G418 stress. Post-injection bleeding and tumor cell escape were avoided by local compression. We measured body weight, blood biochemical indexes and tumor fluorescence every 2 weeks. Anesthetized mice were killed at 16 weeks (intraperitoneal injection of 100 mg/kg ketamine and 10 mg/kg xylazine). This study and all surgery procedures were performed in accordance with the guidelines and regulations of the Care and Use of Laboratory Animals by the US National Academy of Sciences and published by the US NIH (NIH publication 86-23 revised 1985).

### Statistical analysis

All *in vitro* experiments were performed at least in triplicate (unless specified) and 3 parallel samples were measured each time. Comparison of 2 groups involved independent-sample *t* tests (unpaired) for continuous variables and chi-square test for categorical variables. Correlations between variables were determined by the Pearson correlation coefficient. All analyses involved use of SPSS 16.0 (SPSS Inc, Chicago, IL). A two-sided P < 0.05 was considered statistically significant.

## SUPPLEMENTARY MATERIALS FIGURES AND TABLES


