# Supplementary material for: Hyperhomocysteinemia results from and promotes hepatocellular carcinoma via CYP450 metabolism by CYP2J2 DNA methylation
Source: Oncotarget. 2016 Dec 24;8(9):15377–92. doi: 10.18632/oncotarget.14165 (PMC5362492; doi:10.18632/oncotarget.14165)
Supplement: Supplementary file 1 [file oncotarget-08-15377-s001.pdf]

Hyperhomocysteinemia results from and promotes hepatocellular carcinoma via CYP450 metabolism by CYP2J2 DNA methylation

SUPPLEMENTARY FIGURES AND TABLES

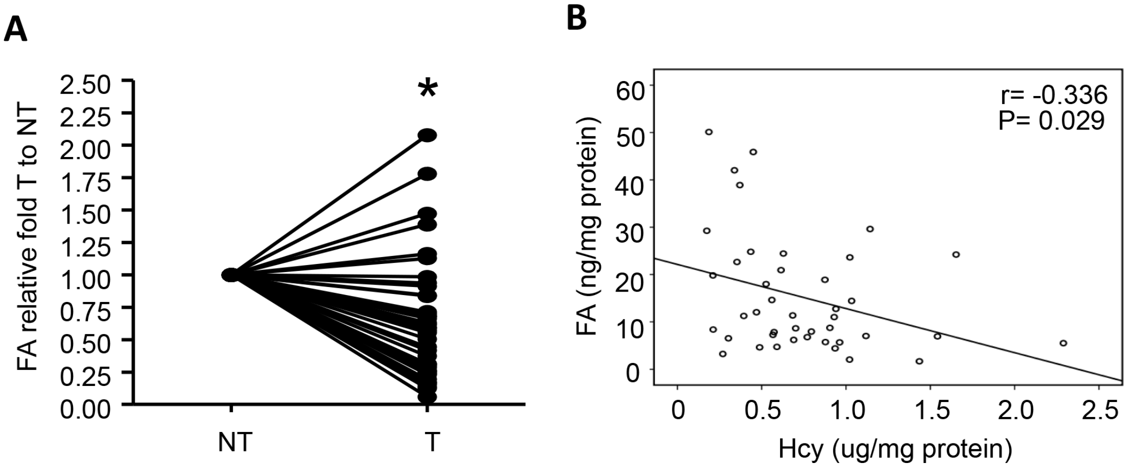

Supplementary Figure 1: Intercellular levels of folic acid (FA) deficiency associated with Increased risk of hepatocellular carcinogenesis. A. Intracellular levels of FA in 42 cases of hepatocellular tumor (T) and adjacent nontumor (NT) tissue samples. **B.** Correlation of FA and homocysteine (Hcy) concentrations in 42 cases of hepatocellular carcinoma tissue. *P<0.05.

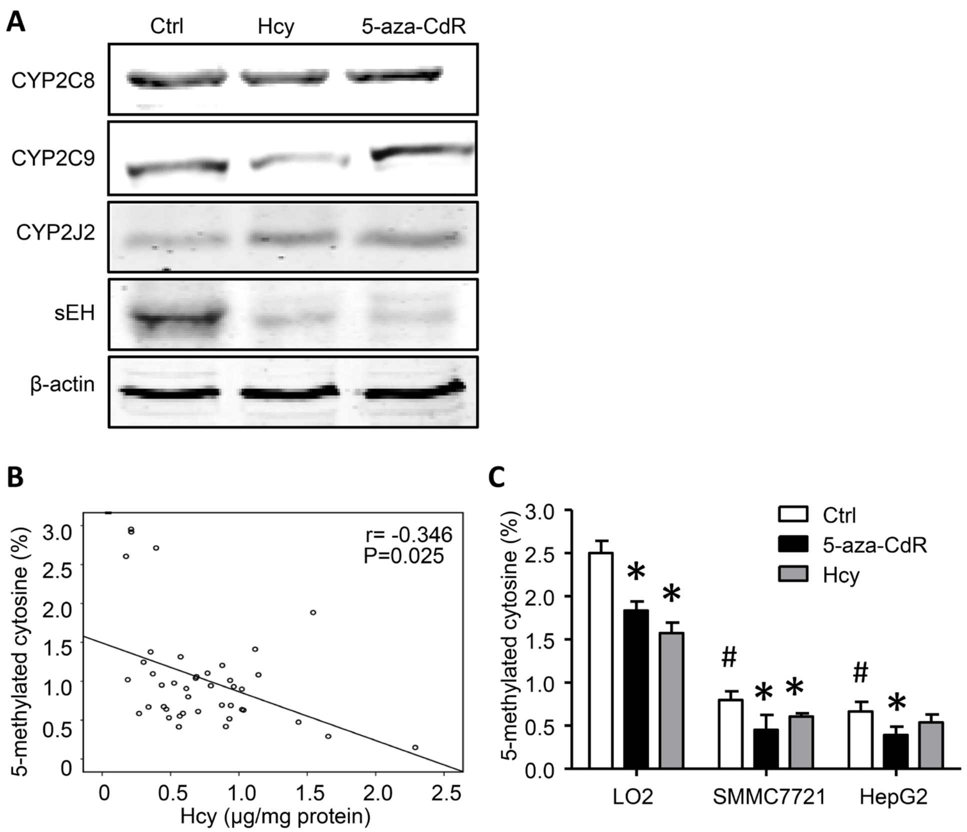

Supplementary Figure 2: A. Western blot analysis of protein levels of CYP2C8, CYP2C9, CYP2J2 and sEH in SMMC7721 cells with Hcy and 5-aza-CdR treatment. β-actin was an internal control. **B.** Correlation of Hcy concentrations and level of 5-methylated cytosine in 42 cases of HCC tissue. **C.** ELISA analysis the changes in level of 5-methylated cytosine in LO2, SMMC7721 and HepG2 cells with the treatment of Hcy and 5-aza-CdR. *P<0.05 vs the corresponding ctrl; #<0.05 vs. ctrl in LO2 cells.

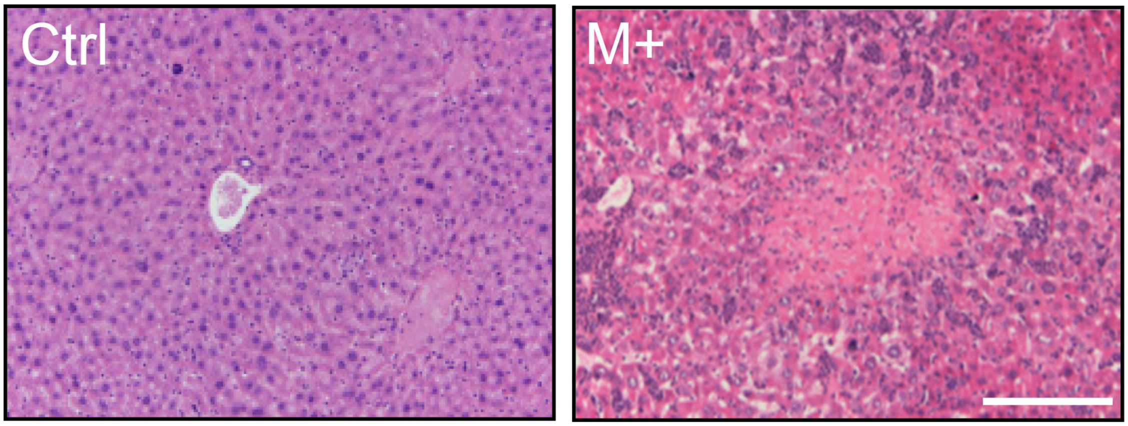

Supplementary Figure 3: Representative images of H&E-stained nude mice with M+ treatment (AIN-93G diet supplemented with L-methionine) for 8 weeks with thicker trabeculae, steatosis and necrosis (400×). Scale bars, 50 µm.

Supplementary Table 1: Epoxyeicosatrienoic acid (EET)-related gene expression in hepatocellular cancerous (HCC) tissue and adjacent non-tumor tissue

Non-tumor tissue			Tumor tissue			P
			-	+	++	
CYP2C8	NT	-	2	2	4	0.911
		+	1	2	7	
		++	5	4	15	
CYP2C9	NT	-	3	4	3	0.271
		+	6	6	4	
		++	3	3	10	
CYP2J2	NT	-	0	8	7	**0.039**
		+	0	9	8	
		++	2	1	7	
sEH	NT	-	10	2	0	**0.037**
		+	6	6	0	
		++	6	8	4	

Sum of frequency and intensity scores: 0 or 1, negative (-); 2 to 3, moderately positive (+); 4 to 6, highly positive (++). sEH, soluble epoxide hydrolase. Data in bold are $P<0.05$.

Supplementary Table 2: Level of clinical characteristics and EET-related gene expression in HCC patients

Characteristic	CYP2J2 protein expression			sEH protein expression		P
	Low (-/+)	High (++)	P	Low (-)	High (+/++)	
Age, years	52.42±9.47	51.95±7.74	0.863	52.10±9.48	52.1±7.64	0.983
Gender, Male/Female	13/7	15/7	0.827	14/8	14/6	0.662
Differentiation High	7	3	**0.016**	2	8	**0.021**
Moderate	10	8		9	9	
Low	3	11		11	3	
Cancer grade I	14	9	0.071	11	12	0.887
II	3	10		7	6	
III	3	3		4	2	
Tumor sizes	63.80±93.08	237.27±286.53	**0.014**	237.60±301.91	79.27±99.87	**0.025**
AFP (U/L)	67.39±73.42	326.17±432.76	**0.01**	226.48±346.80	181.54±328.67	0.669
ALT (U/L)	55.90±54.06	83.83±7.70	0.183	60.35±48.43	79.33±79.36	0.361
AST (U/L)	47.26±31.37	71.98±69.37	0.151	47.23±29.19	72.01±70.22	0.140
Smoking, Yes/No (no.)	15/5	14/8	0.332	13/9	16/4	0.143
Drinking, Yes/No (no.)	10/10	14/8	0.445	10/12	14/6	0.108
Family history, Yes/No (no.)	15/5	16/6	0.867	18/4	13/7	0.216
HBV, Yes/No (no.)	7/13	10/12	0.491	11/11	6/14	0.187
HCV, Yes/No (no.)	3/17	3/19	1.00	4/18	2/18	0.665
sEH relative mRNA	0.78±0.72	0.60±0.80	0.448	0.40±0.32	0.94±0.94	**0.019**
CYP2J2 relative mRNA	1.55±3.48	22.71±44.69	**0.041**	8.33±23.60	16.55±41.16	0.438
CYP2C8 relative mRNA	1.75±3.24	3.31±4.59	0.214	2.32±3.01	2.79±4.85	0.712
CYP2C9 relative mRNA	4.11±9.88	4.87±8.81	0.795	3.19±7.48	5.71±10.60	0.383
HCY (μmol/L)	0.65±0.39	1.42±1.25	**0.013**	1.22±1.19	0.90±0.811	0.306
FA (ng/mL)	22.39±14.94	14.84±11.30	0.071	12.02±10.55	24.02±13.75	**0.004**

AFP, alpha-fetoprotein; AST, aspartate aminotransferase; ALT, alanine aminotransferase; HBV and HCV, hepatitis B and C virus; HCY, homocysteine; FA, folic acid. Data in bold are P<0.05.

Supplementary Table 3: Clinical features of BALB/c nude mice fed diets varying in methionine and B vitamins for 8 weeks

Factors	Ctrl	M+	M+ F+	M+F-
Hcy level (μmol/L)	4.25±0.79	**54.74±5.86[a]**	**27.17±7.86[b]**	**85.89±7.30[a]**
Folate (ng/mL)	74.01±16.56	80.01±14.81	**136.50±27.19[b]**	**42.13±17.58[a]**
Vitamin B12 (pg/ml)	9825.98±1697.12	**6446.45±1292.07[a]**	**28902.54±3676.44[b]**	**3723.51±1649.91[a]**
ALT (U/L)	41.60±6.54	45.93±9.68	42.67±8.22	49.80±7.02
AST (U/L)	146.37±15.03	156.22±40.91	141.68±33.01	**172.97±37.42[a]**
ALP (U/L)	16.70±5.33	21.68±6.47	16.37±6.60	**29.57±13.34[a]**
TBA (μmol/L)	0.81±0.32	**1.29±0.48[a]**	1.05±0.16	1.55±1.00
TC (mmol/L)	2.55±0.62	**3.43±0.40[a]**	3.26±0.61	**3.41±0.45[a]**
TG (mmol/L)	0.49±0.21	0.69±0.15	0.50±0.24	0.78±0.80
HDL (mmol/L)	2.82±0.72	2.25±0.27	2.24±0.36	**2.14±0.22[a]**
LDL (mmol/L)	0.14±0.03	**0.21±0.06[a]**	0.19±0.06	**0.27±0.08[a]**
Apo B (g/L)	0.06±0.02	0.08±0.01	0.08±0.01	0.08±0.02
CRP (ng/mL)	0.06±0.03	**0.10±0.02[a]**	**0.07±0.03[b]**	**0.12±0.03[a]**

F, folic acid; AST, aspartate aminotransferase; ALT, Alanine aminotransferase; ALP, alkaline phosphatase; TBA, total bile acids; TC, total cholesterol; TG, triglycerides; HDL, LDL, high and low density lipoprotein; Apo B, apolipoprotein B; CRP, C-reactive protein level. Data in bold are $P<0.05$.
